# Supplementary material for: Using synchronized oscillators to compute the maximum independent set
Source: Nat Commun. 2020 Sep 17;11:4689. doi: 10.1038/s41467-020-18445-1 (PMC7499257; doi:10.1038/s41467-020-18445-1)
Supplement: Supplementary file 1 — Supplementary Information [file 41467_2020_18445_MOESM1_ESM.pdf]

Supplementary Information

Using Synchronized Oscillators to Compute the Maximum Independent Set

Antik Mallick[1], Mohammad Khairul Bashar[1], Daniel S. Truesdell[1], Benton H. Calhoun[1], Siddharth Joshi[2], and Nikhil Shukla[1]

[1]Department of Electrical and Computer Engineering, University of Virginia, Charlottesville, Virginia 22904, USA

[2]Department of Electrical and Computer Engineering, University of Notre Dame, Notre Dame, IN 46556, USA.

Correspondence to ns6pf@virginia.edu

Supplementary Figures

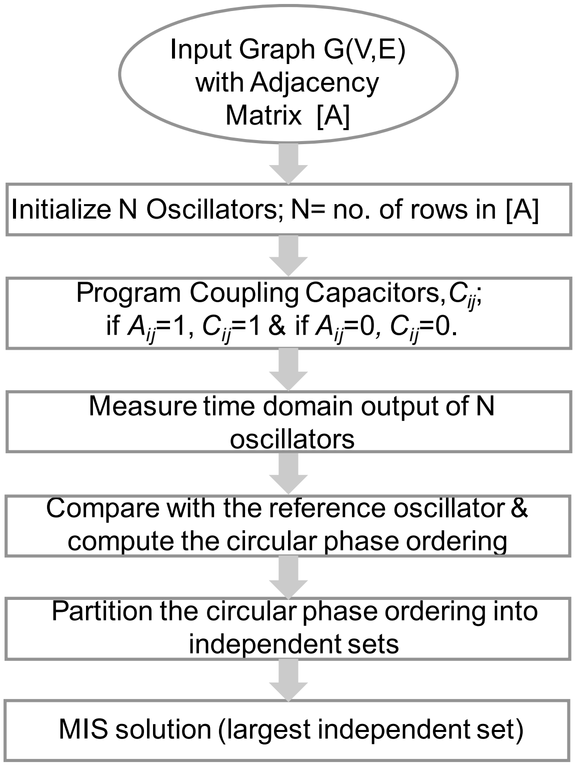

Supplementary Figure 1| Process flow for computing the MIS using the coupled oscillators.

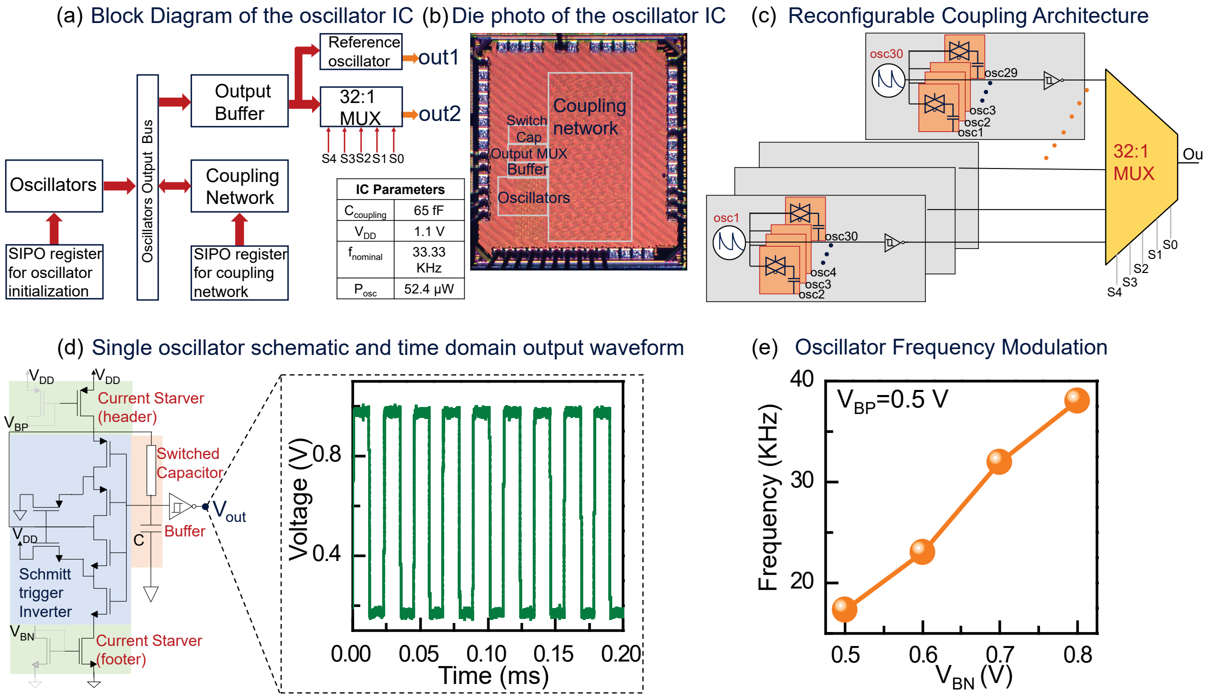

Supplementary Figure 2| Coupled Oscillator Platform (a) Block diagram; (b) Die photo of the IC consisting of coupled oscillators. (c) Schematic of the reconfigurable capacitive coupling scheme. The design facilitates 'all-to-all' connectivity which enables any arbitrary graph (up to 30 nodes) to be processed using the oscillators. (d) Circuit schematic and time domain output waveform of a single oscillator. (e) Programmability of the oscillator frequency using the current starver circuit.

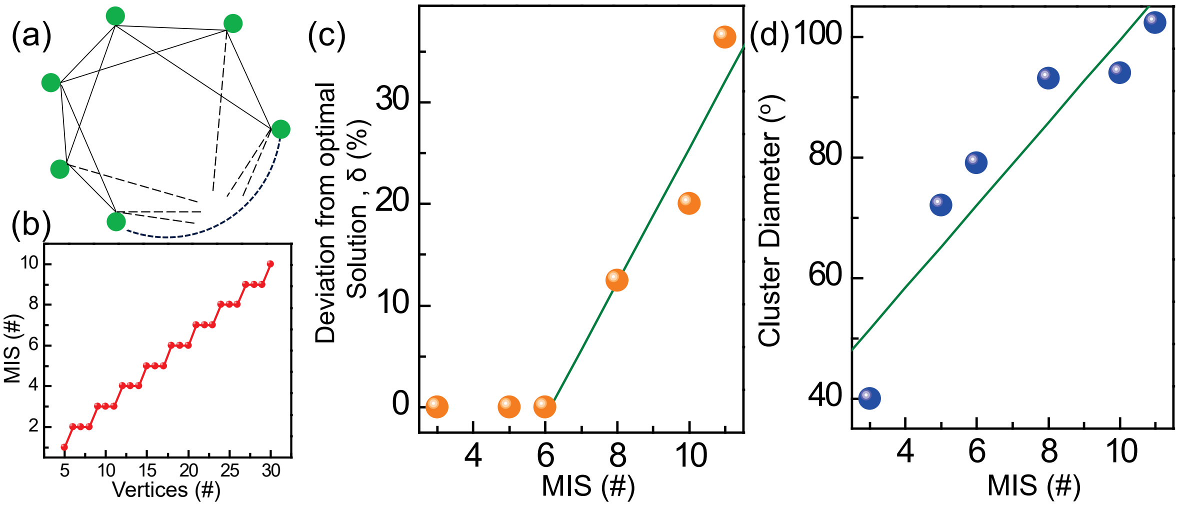

Supplementary Figure 3| Computing MIS in nearest neighbor graphs using coupled oscillators. (a) Schematic; and (b) MIS as function of graph size for k (=4) nearest neighbor connected graphs. Evolution of (c) deviation from optimal solution computed by the oscillators; and (d) cluster diameter as a function of MIS size.

(a)

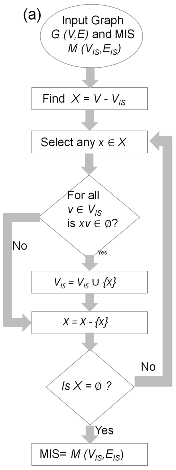

(b)

DIMACS Graphs					
Graph	Node	Avg. Connectivity	MIS w/o post-processing	MIS w/ post-processing	MIS from B-K Algorithm
2dc_128	128	0.63	4	5	5
1et_64	64	0.13	12	18	18
1dc_64	64	0.27	8	10	10
2dc_256	256	0.52	5	6	7
1dc_128	128	0.18	9	16	16
1tc_32	32	0.13	12	12	12
1tc_64	64	0.09	14	20	20
1zc_128	128	0.28	12	18	18
1et_128	128	0.083	17	28	28

(c)

Random Graphs				
Node	Avg. Connectivity	MIS w/o post-processing	MIS w/ post-processing	MIS from B-K Algorithm
64	0.2	9	16	17
64	0.2	10	18	18
64	0.2	9	16	16
64	0.4	6	10	10
64	0.4	6	10	10
64	0.4	6	9	9
64	0.6	4	6	6
64	0.6	5	7	7
64	0.6	4	7	7
64	0.8	4	4	5
64	0.8	3	5	5
64	0.8	3	4	5
128	0.2	9	21	No solution after 80 hours
128	0.2	9	20	No solution after 80 hours
128	0.2	9	20	No solution after 80 hours
128	0.4	6	11	12
128	0.4	6	11	12
128	0.4	6	11	13
128	0.6	5	7	8
128	0.6	5	7	8
128	0.6	5	7	8
128	0.8	3	5	5
128	0.8	3	4	5
128	0.8	4	5	5
160	0.2	10	20	No solution after 80 hours
160	0.2	9	21	No solution after 80 hours
160	0.2	11	21	No solution after 80 hours
160	0.4	6	10	13
160	0.4	6	11	13
160	0.4	7	11	13
160	0.6	5	8	8
160	0.6	4	8	9
160	0.6	5	7	9
160	0.8	3	5	5
160	0.8	3	5	6
160	0.8	3	5	5

(d)

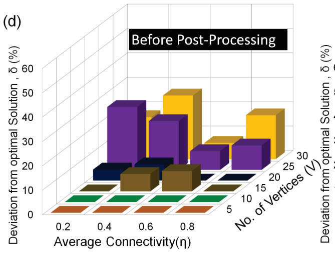

(e)

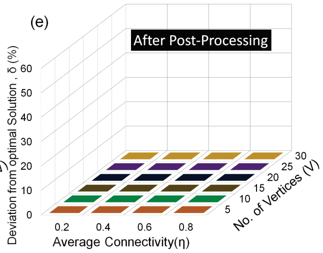

Supplementary Figure 4| Post-processing to improving MIS solution. (a) Flow chart for the proposed post-processing scheme to improve the MIS solution obtained from the oscillators in larger graphs. (b)(c) Corresponding improvement in the MIS solution analyzed in DIMACS implementation challenge graphs and simulated random graph instances, respectively; (d)(e) Improvement in MIS solution for the experimentally measured graphs in the main text.

Coupled oscillator   Heuristic algorithm   Bron-Kerbosch (B-K) algorithm

Average Connectivity (128 node)	MIS Solution	Time to Compute	Remark
0.2	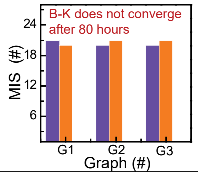	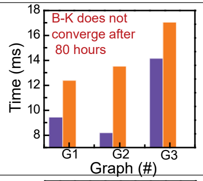	• **Comparison with B-K algorithm**: No convergence observed after 80 hours.<br>• **Comparison with B-K algorithm**: 26.82% average reduction in computing time.
0.4	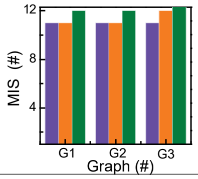	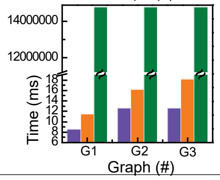	• **Comparison with B-K algorithm**: 10^6x improvement in average time to compute.<br>• **Comparison with B-K algorithm**: 26.04% average reduction in computing time.
0.6	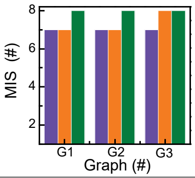	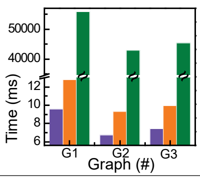	• **Comparison with B-K algorithm**: 10^3x improvement in average time to compute.<br>• **Comparison with B-K algorithm**: 25.9% average reduction in computing time.
0.8	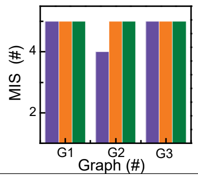	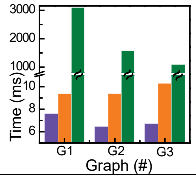	• **Comparison with B-K algorithm**: 10^2x improvement in average time to compute<br>• **Comparison with B-K algorithm**: 28.18% average reduction in computing time.

Supplementary Figure 5| Comparison with other computational approaches. Comparison of MIS solution and required computing time for B-K , heuristic algorithm and the coupled oscillators for 128 node graphs with η=0.2-0.8; three graphs are considered for each η. The oscillator computing time includes time for the system to reach steady-state as well as the post-processing scheme

Supplementary Notes

Supplementary Note 1

Process flow for computing the maximum independent set (MIS). Supplementary Figure 1 shows the process flow for computing the maximum independent set (MIS) of an input graph using the coupled oscillator platform. Initially, the oscillator chip is programmed to represent the input graph based on the adjacency matrix; A_{ij}=1 (0) enables (disables) the capacitive coupling between i & j (represented by oscillators in the hardware) by turning ON (OFF) the corresponding transmission gate. Once the oscillators synchronize, the time domain waveform of each oscillator is measured serially using the MUX. The output of the oscillators is compared with the output of a reference oscillator, and is subsequently, used to calculate the phase difference and the circular phase ordering sequence. The phase sequence has a unique property where vertices belonging to an independent set appear adjacent to each other (described below). Consequently, the vertices of the graph can then be partitioned into independent sets through a simple sorting operation. The largest of the independent set represents an optimal / near-optimal solution of the MIS.

Phase ordering of the oscillators and its relationship to independent sets of the graph. The maximum independent set (MIS) of a graph is defined as the largest set of nodes, no two of which are connected. Mathematically, a set $S_{IS} = \{V_{IS}, E_{IS}\}$ is an independent set, if $E_{IS} = \emptyset$. The largest possible $S_{IS} = \{V_{IS}, E_{IS}\}$ is then the MIS.

To elucidate how the phase characteristics of the oscillators facilitate the computation of the MIS, we consider the system dynamics of the oscillators. As described above, we represent each node as an oscillator and every edge as a coupling capacitor. This implies that when: A_{ij} = 1, C_{ij} = C_C; A_{ij} = 0, C_{ij} = 0. For a coupled system of n oscillators, the current through any oscillator k can be described as:

$$I_k = (C_{ik})x_k' + \sum_{j=1}^{n} A_{kj} C_c (x_k' - x_j') + g_{ik} x_k \quad (1)$$

where I_k, x_k, g_i, C_i is the output current, output voltage, internal capacitance and internal conductance of kth oscillator, respectively. Furthermore, this system can be represented in state-space as:

$$X' = BX + C \quad (2)$$

where $X = [x_1\ x_2\ ...\ x_n]^T$ and $B = g_i(C_cA - (C_i + nC_c)I)^{-1}$.

The eigenvalues of B are related to the oscillator phases (states). These can be described by the relative temporal position of their peaks which essentially represents a circular phase ordering[1]. Furthermore, it can be shown that the eigenvalues of B are directly related to the eigenvalues of the adjacency matrix, A[1]. Consequently, the eigenvalues of A can be mapped to the phase ordering of the oscillators. Using spectral graph theory[1-3], it can be shown that vertices of an independent set have comparable eigenvalues of A. Further, if in the eigen-spectrum when large changes in the eigenvalues are observed, the corresponding vertices belong to a different independent set. Consequently, this is reflected in the phase ordering of the coupled oscillators as well. This ordering can subsequently be partitioned using a simple sorting scheme (with time complexity of $O(n^2)$) to compute the near-optimal/optimal MIS.

Supplementary Note 2

Integrated circuit (IC) design for reconfigurable coupled oscillator platform. The IC used to implement and characterize the computational properties of the coupled oscillators is fabricated using the bulk CMOS 65nm technology and occupies a total area of 1.44 mm^2. The measured power dissipation of the chip is 1.7 mW with each oscillator consuming 52.4 µW. Supplementary Figure 2a shows the block diagram of the IC which incorporates the following:

1. Relaxation Oscillator. The basic computational unit of our hardware platform is the relaxation oscillator which is implemented using a Schmitt trigger inverter module along with a negative RC feedback (Supplementary Figure 2d). The total area occupied by the oscillator is approximately 28 µm^2. The oscillator implementation utilizes:

A. Switched Capacitor: The feedback resistor (R_F = 402 MΩ) is implemented using a switched capacitor. The target resistance is achieved by applying two out-of-phase

sinusoidal signals of frequency (f) 1.5 MHz onto the gates of the two MOSFETs that connects the capacitor (C_{gg} = 1.6 fF) to the input and output of the switched capacitor circuit; $R_F = 1/(f*C_{gg})$.

B. Output Buffer: The relaxation-type output of each oscillator is passed through an inverting hysteretic output buffer which digitizes the output while preserving the critical phase information (Supplementary Figure 2d). The binary output of the oscillator simplifies the measurement and read-out of the oscillator phases which is essential for computing the MIS.

C. Current starver circuit: The operating parameters of the oscillator such as frequency can be controlled using the current starver circuits implemented at the header and the footer. The current starver helps modulate the charging and discharging current of the oscillator and therefore, the oscillation frequency as shown in Supplementary Figure 2e.

2. Reconfigurable Coupling Architecture. Figure Supplementary Figure 2c shows a detailed view of the "all-to-all" reconfigurable coupling scheme. Each oscillator can be coupled to any other and all other oscillators through a T-gate and a total coupling capacitance (= 65fF). The coupling elements are programmed according to the adjacency matrix of the input graph. When there is an edge between two vertices i.e. $A_{ij} = A_{ji}$= '1', the corresponding T-gate associated with the coupling capacitor $C_{ij}=C_{ji}$=32.5 fF is switched ON to facilitate capacitive coupling between vertices i and j. We note that to realize a complete graph (with full reconfigurability), the size of the coupling capacitor array (corresponding to the number of edges) has a square-law dependence on the number of vertices (oscillators) $E \sim n^2$. However, in practical graphs which are usually sparse ($E \sim n$)[4], this constraint can be relaxed to reduce the coupling capacitor array size without putting significant constraints on the graphs that can be solved by the hardware.

3. I/O Ports

A. Serial-input parallel-output (SIPO) shift registers: Serial-input, parallel-output (SIPO) shift registers are used to program the oscillators (ON/OFF) and the coupling network.

B. 32:1 MUX: A 32:1 Multiplexer is used at the output to select and read the time domain output waveform of each oscillator in a serial fashion. The outputs are read using a PGLA (Pattern Generator Logic Analyzer).

Supplementary Note 3

Computational properties of oscillators analyzed using K-nearest (K=4) neighbor connected graphs. We analyze using simulations, the evolution of cluster diameter and optimality of the solution in k-nearest-neighbor connected graphs (k=4) of varying size wherein every node is connected to its 4 nearest neighboring nodes (Supplementary Figure 3a); the corresponding MIS is shown in Supplementary Figure 3b. Unlike the randomly generated graphs considered in the main text, these graphs maintain the same underlying connectivity pattern. It can be observed from Supplementary Figure 3c, d that both the accuracy (quantified as deviation from optimal solution) and the cluster diameter increase with the size of the MIS, following the same trends as seen in Fig. 3 (main text) albeit with a smaller deviation.

Supplementary Note 4

Post-processing Scheme to Improve the Quality of MIS Solution. The simulations for evaluating the MIS in larger graphs are performed using the Xyce platform – an open-source, SPICE-compatible, high-performance analog circuit simulator offered Sandia National Labs[5]. The Xyce simulator is interfaced with MATLAB which is used to input the circuit simulation parameters required for Xyce as well as to evaluate the output obtained from the circuit simulation. The CMOS Schmitt trigger-based oscillator circuit is built in Xyce using the 65nm BSIM model[6]. We intentionally introduce mismatch among the oscillators using variations in the R and C values in the feedback circuit. In order to find

the MIS solution of an input graph, the adjacency matrix of the graph is provided to MATLAB which subsequently, programs the oscillator and the corresponding capacitive coupling among them. The oscillator dynamics are then simulated on Xyce for a maximum of 1 ms with a timestep of 0.1 μs wherein we observe that the oscillators always attain steady-state for the simulated graphs. The time-domain output from the oscillators is then exported to MATLAB. The relative oscillator phase difference in steady-state is used to construct the circular phase ordering for computing the MIS solution (as illustrated in Supplementary Note 1). Finally, a proposed post-processing scheme as described below is used to improve the MIS solution by expanding the largest independent set (obtained from the oscillator ordering).

We evaluate the scalability of the proposed approach to compute the MIS in larger graphs that exceed (the experimentally available) thirty nodes. We observe that the MIS solution is sub-optimal in some instances wherein certain vertices of the optimal MIS appear as a part of a different independent set, and consequently, are excluded from the optimal MIS solution. This can be attributed to the reduced phase separation between the oscillators where the small rounding errors can disturb the optimal ordering sequence; in an experiment, the small phase separation along with noise is likely to lead to errors in reading out the exact output phase.

However, we observe that the quality of the MIS solution can be dramatically improved by executing a simple (heuristic) post-processing step that has polynomial (*$O(n^2)$*) time complexity. It entails expanding the largest independent set (obtained from the oscillator ordering) to ensure that it is maximal. This illustrated in the flowchart shown in Supplementary Figure 4a.

Let $M = (V_{IS}, E_{IS})$ be the largest IS obtained from the dynamics of the coupled oscillators for an input graph $G = (V, E)$. A node $x \in X = V - V_{IS}$ is added to M, if $\forall\ v \in V_{IS}$, any edge $xv \in \emptyset$. Subsequently, X is updated to $X - x$. This process is repeated for all $x \in X$ until M is a maximal independent set. If V and V_{IS} has n and m elements respectively, the expansion requires $(m-1) \times n$ operations. Since $m < n,$ the number of operations required (in the worst-case) is $(m-1) \times n < n^2$.

Supplementary Figure 4b,c compares the MIS solution before (obtained from the oscillators) and after applying the post-processing step for the DIMACS graphs (Fig. 4b of main text), and the simulated random graph instances (in Fig. 4a of main text), respectively. Similarly, Supplementary Figure 4 d,e show the improvement in the MIS solution for the experimentally measured graphs after implementing the post-processing step.

Supplementary Note 5

Comparison of the Oscillator Approach with other Approaches to Compute MIS. We compare using simulations the coupled oscillator based computing approach for solving MIS with both the heuristic[7] and the exact algorithm (Bron-Kerbosch algorithm) for randomly generated 128 node graphs across a wide range of connectivity (η=0.2, 0.4, 0.6, 0.8). It can be observed from Supplementary Figure 5 that the oscillators (including post-processing) provide 25-28% improvement in computing time compared to the heuristic approach while providing similar solutions. In comparison to the B-K algorithm (which produces an optimal solution if it converges), the oscillators can provide 10^2-$10^6\times$ speedup albeit with a small deviation from optimal MIS.

Supplementary References

1. Parihar, A., Shukla, N., Jerry, M., Datta, S., & Raychowdhury, A. Vertex coloring of graphs via phase dynamics of coupled oscillatory networks. *Scientific reports* **7,** 911 (2017).

2. McSherry. F. Spectral partitioning of random graphs. *In 42nd IEEE Symposium on Foundations of Computer Science*, 529–537 (IEEE, Newport Beach, CA, 2001).

3. Aspvall, B. and Gilbert, J.R. Graph coloring using eigenvalue decomposition. *SIAM Journal on Algebraic Discrete Methods* **5,** 526-538 (1984).

4. Liu, G. and Zhang, Z., 2015, June. A reconfigurable analog substrate for highly efficient maximum flow computation. In *Proceedings of the 52nd Annual Design Automation Conference* (pp. 1-6).

5. Keiter, E.R., et al. Xyce parallel electronic simulator release notes. *No. SAND2015-3379. Sandia National Lab.(SNL-NM), Albuquerque, NM* (United States); Raytheon, Albuquerque, NM, 2015.

6. Predictive Technology Model, http://ptm.asu.edu/.

7. Balaji, S., Swaminathan, V. and Kannan, K. A simple algorithm to optimize maximum independent set. *Advanced Modeling and Optimization* ***12***, 107-118 (2010).
